# Autoimmune pancreatitis limited to the main pancreatic duct without mass formation

**DOI:** 10.1007/s12328-026-02315-2

**Published:** 2026-03-27

**Authors:** Kaoruko Kanamaru, Arata Sakai, Takashi Kobayashi, Atsuhiro Masuda, Norimitsu Uza, Takayuki Kodama, Yoshihide Nanno, Takumi Fukumoto, Yuzo Kodama

**Affiliations:** 1 Division of Gastroenterology, Department of Internal Medicine, Kobe University Graduate School of Medicine, 7-5-1 Kusunoki- cho, Chuo-ku, Kobe, Hyogo 650-0017 Japan; 2 Division of Molecular and Genomic Pathology, Department of Pathology, Kobe University Graduate School of Medicine, 7-5-1 Kusunoki-cho, Chuo-ku, Kobe, Hyogo 650-0017 Japan; 3 Division of Hepato-Biliary-Pancreatic Surgery, Department of Surgery, Kobe University Graduate School of Medicine, 7-5-2 Kusunoki-cho, Chuo-ku, Kobe, Hyogo 650-0017 Japan

**Keywords:** Autoimmune pancreatitis, Main pancreatic duct, Carcinoma in situ, Case report

## Abstract

We present a rare case of autoimmune pancreatitis (AIP) confined exclusively to the subepithelial layer in the main pancreatic duct (MPD) without associated pancreatic enlargement or mass formation. A 73-year-old man presented with elevated serum pancreatic enzymes and IgG4 levels. Imaging revealed a short-segment MPD stenosis in the body of the pancreas without pancreatic swelling. Endoscopic ultrasonography showed localized periductal thickening of the MPD, raising suspicion of pancreatic carcinoma in situ (CIS). Cytological analysis of pancreatic juice identified atypical cells, prompting surgical resection. Histopathological examination of the resected specimen revealed characteristic features of type 1 AIP, including dense lymphoplasmacytic infiltration, storiform fibrosis, and obliterative phlebitis, which were strictly confined to the subepithelial layer in the MPD. No malignant cells were found, and the surrounding pancreatic parenchyma showed only minimal fibrotic changes. This case may broaden the recognized clinical spectrum of AIP by demonstrating a rare presentation without mass formation or pancreatic swelling, which closely mimicked pancreatic CIS. Further accumulation of cases may enhance our comprehensive understanding of this disease entity.

## Introduction

Autoimmune pancreatitis (AIP) is a subtype of pancreatitis that is linked to autoimmune processes. Histologically, it is characterized by dense infiltration of lymphocytes and plasma cells accompanied by fibrosis [1]. Radiologically, AIP typically manifests as diffuse or focal enlargement of the pancreas [2].

Focal-type AIP is challenging in clinical settings because its imaging characteristics closely mimic those of pancreatic ductal adenocarcinoma (PDAC). Moreover, focal AIP manifests as a mass-forming lesion accompanied by segmental ductal stenosis, making it challenging to differentiate from PDAC and potentially leading to unnecessary surgery. Recent developments, such as EUS-guided fine-needle aspiration, have improved the accuracy of histological diagnosis [3]. Herein, we present an exceptionally rare case of AIP, in which the distinctive pathological features, including storiform fibrosis, lymphoplasmacytic infiltration, and IgG4-positive plasma cells, were restricted to the subepithelial layer in the MPD, with no involvement of the parenchyma or mass formation.

## Case report

**Fig. 1:**
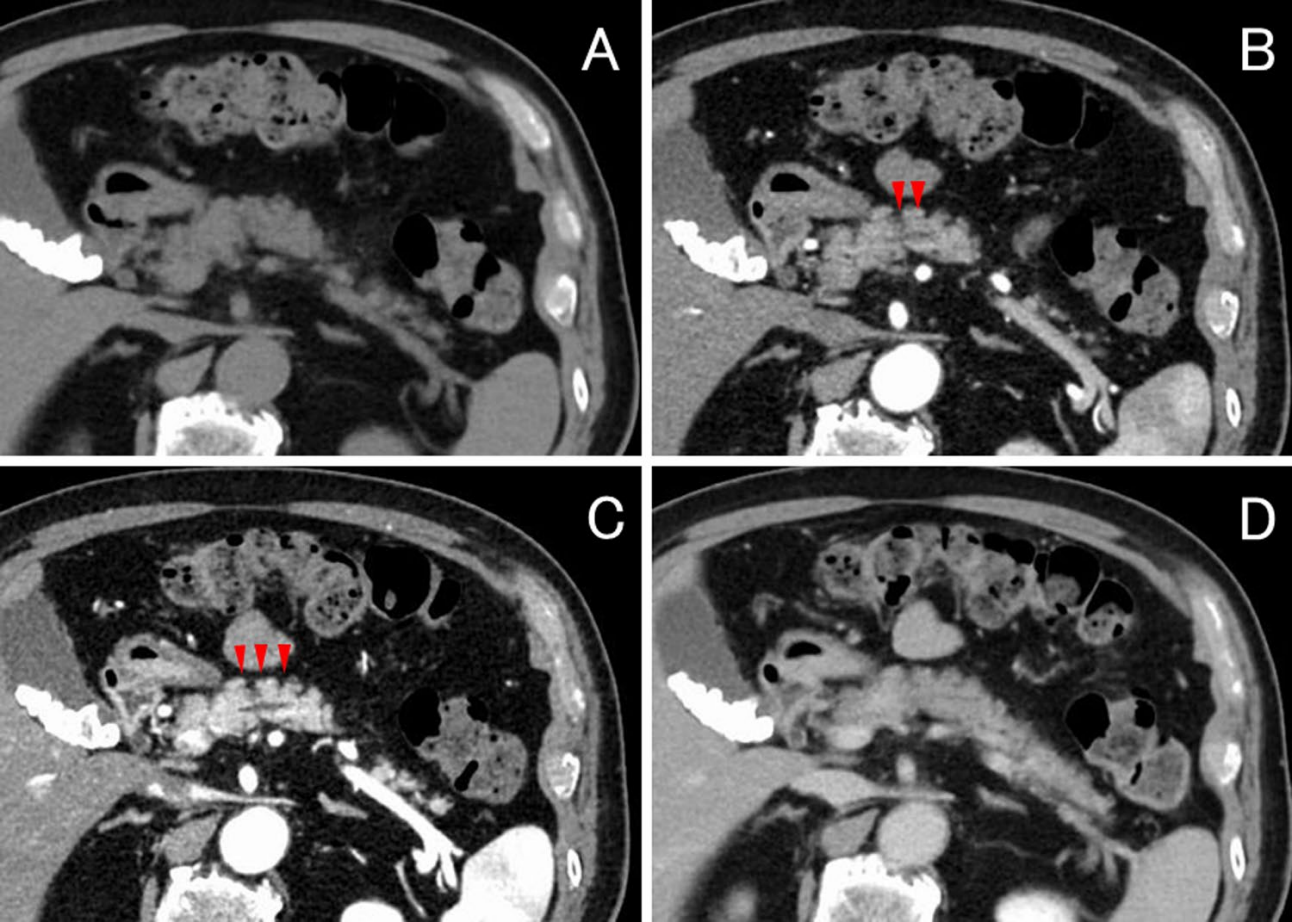
Contrast-enhanced computed tomography of the pancreas in four phases: **A** Unenhanced phase, **B** Arterial phase, **C** Portal venous phase, and **D** Equilibrium phase. The images show mild dilation of the main pancreatic duct in the body of the pancreas (red arrows), without any detectable mass lesion

**Fig. 2:**
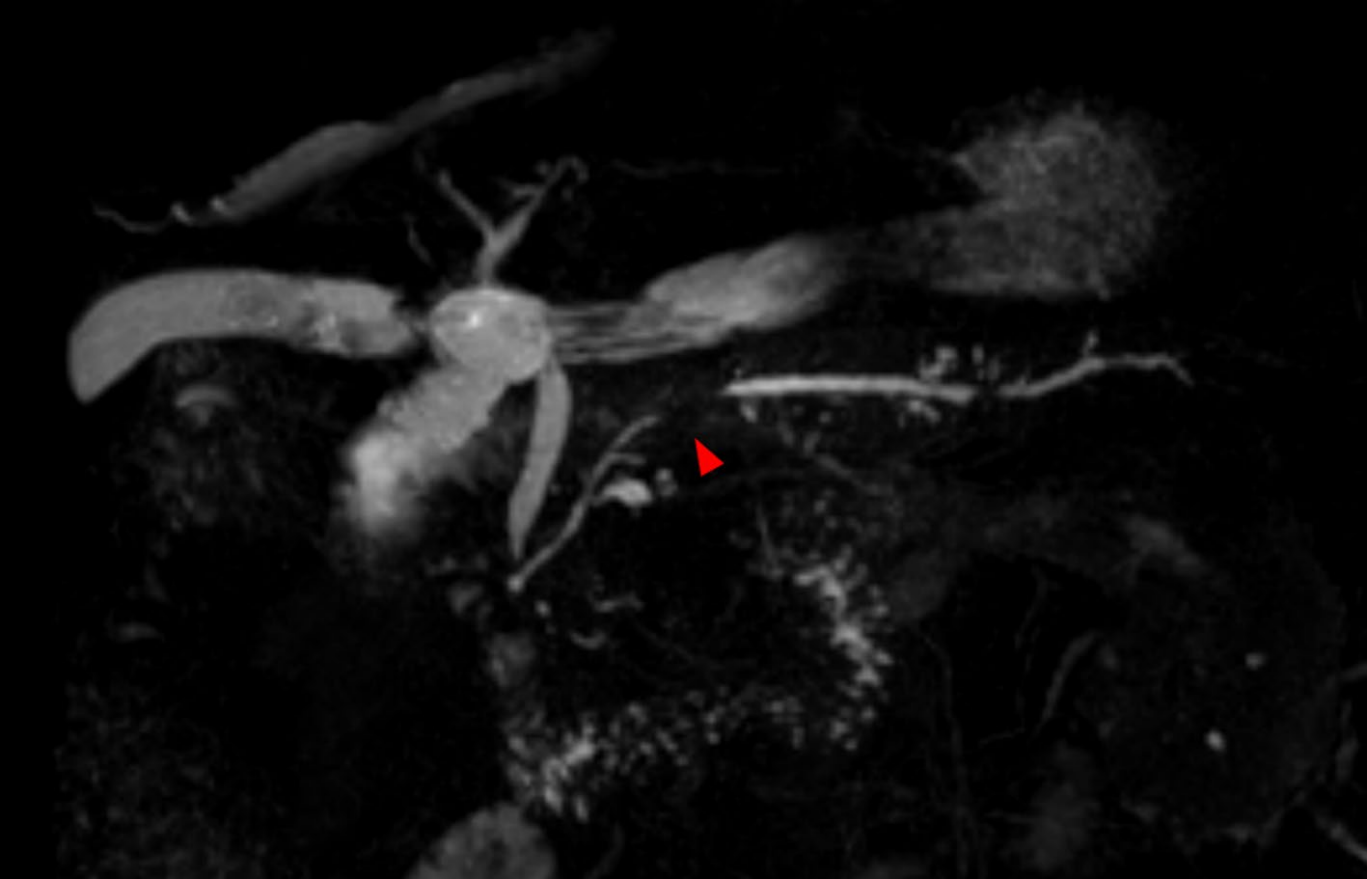
Magnetic resonance cholangiopancreatography reveals a 10 mm segmental stenosis of the main pancreatic duct (MPD) in the pancreatic body, accompanied by mild dilatation of the upstream MPD and its branch ducts. The area of stenosis is indicated by red arrows

**Fig. 3:**
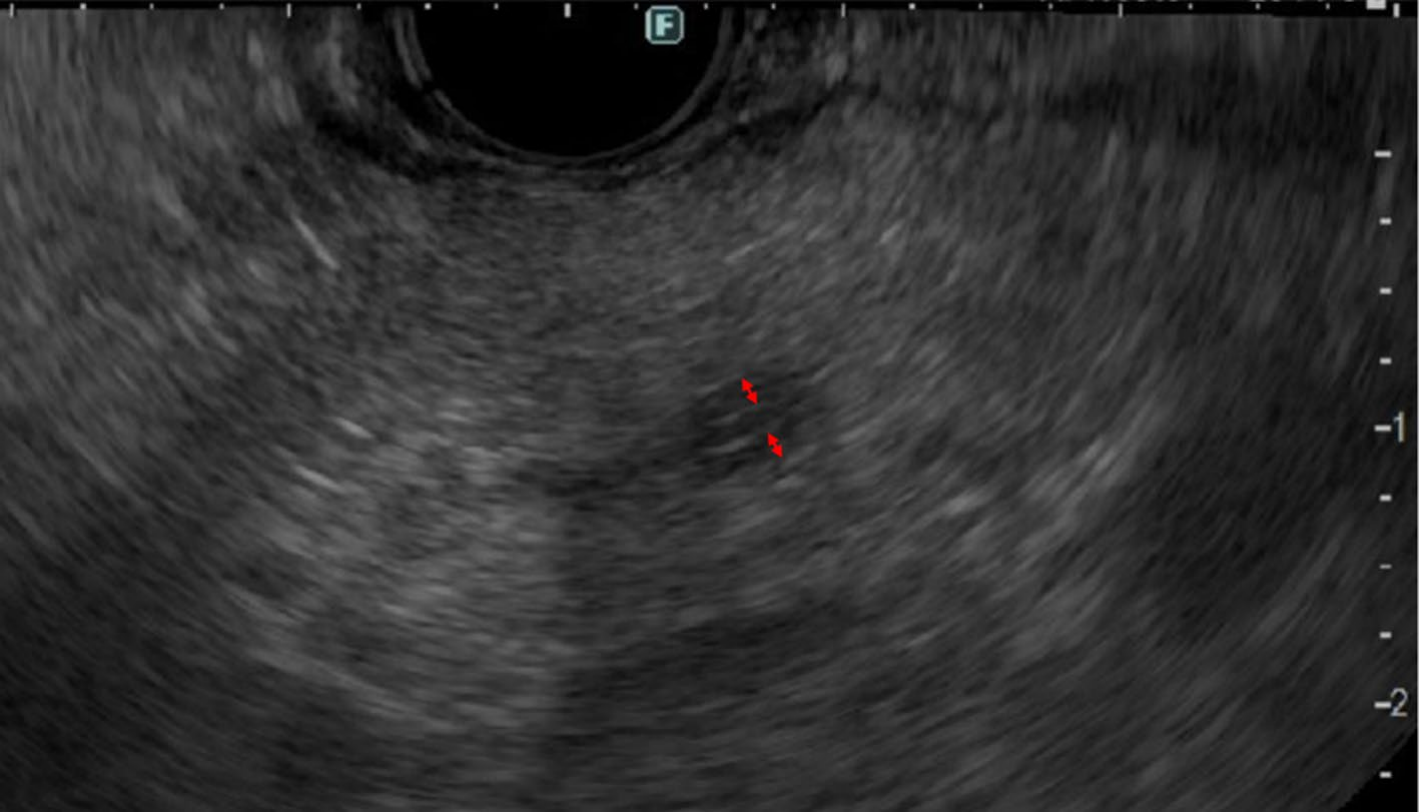
Endoscopic ultrasound showed no evidence of pancreatic parenchymal enlargement or mass lesion. However, the main pancreatic duct in the pancreatic body was sharply dilated to approximately 3 mm, with localized mural thickening. The site of ductal dilatation and mural thickening is indicated by double-headed arrows

**Fig. 4:**
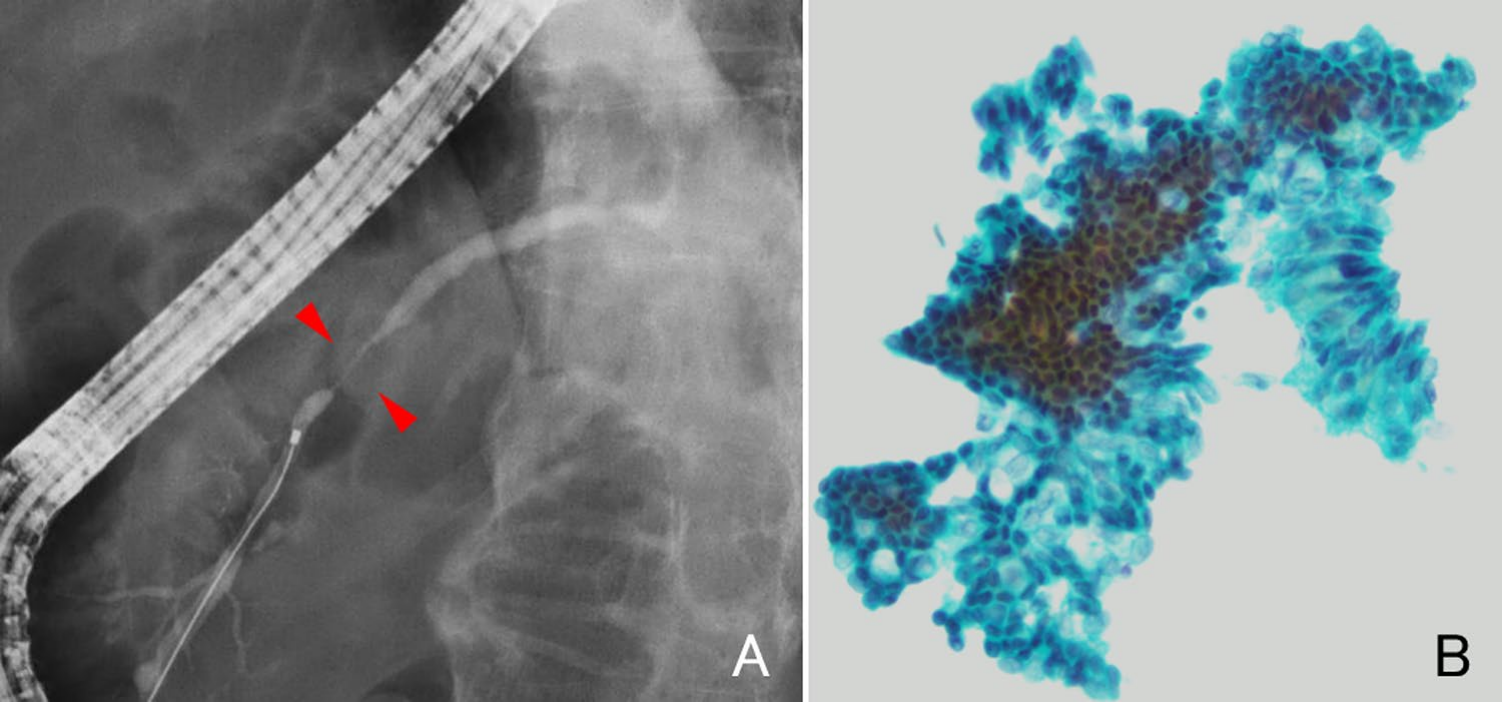
**A** Endoscopic retrograde cholangiopancreatography demonstrates a 10-mm focal stenosis of the main pancreatic duct (MPD), as well as slight dilatation of the upstream MPD. The site of ductal stenosis is indicated by red arrows. **B** Brush cytology and pancreatic juice cytology from the stenotic segment showing mildly atypical cells with nuclear enlargement, irregular contours, and a high N/C ratio, raising suspicion of malignancy. Papanicolaou stain

A 73-year-old man was referred to our hospital following an incidental finding of elevated pancreatic enzyme levels during a routine health checkup. Laboratory tests revealed elevated amylase (55 U/L; 16–52), lipase (100 U/L; 16–60), and serum IgG4 (295 mg/dL; 11–121). Tumor markers were within normal ranges: CA19-9 < 1 U/mL (< 37), CEA 0.8 ng/mL (< 5), DUPAN-2 34 U/mL (< 150), and Span-1 3 U/mL (< 30). Contrast-enhanced CT revealed mild dilation of the MPD in the body of the pancreas, but no mass was observed (Fig. 1). Magnetic resonance cholangiopancreatography (MRCP) revealed a 10 mm segmental stenosis of the MPD in the pancreatic body, with mild dilatation of the upstream MPD and branch ducts (Fig. 2). EUS revealed no signs of pancreatic parenchymal enlargement or mass lesions; however, the MPD in the pancreatic body was distinctly dilated (approximately 3 mm) and accompanied by symmetric band-like hypoechoic areas along the MPD (Fig. 3). Based on these findings, pancreatic carcinoma in situ (CIS) was suspected, and endoscopic retrograde cholangiopancreatography (ERCP) was performed for further evaluation. ERCP also demonstrated a 10 mm focal MPD stenosis and slight dilatation of the upstream MPD (Fig. 4A). Cytological examinations, including brush cytology and pancreatic juice aspiration cytology from the stenotic segment, revealed scattered cells with nuclear enlargement and irregular nuclear contours, as well as a high nuclear-to-cytoplasmic ratio, raising suspicion of malignancy (Fig. 4B). However, the number of atypical cells was limited and the degree of nuclear atypia was relatively mild, making it difficult to definitively differentiate between reactive and malignant changes.

**Fig. 5:**
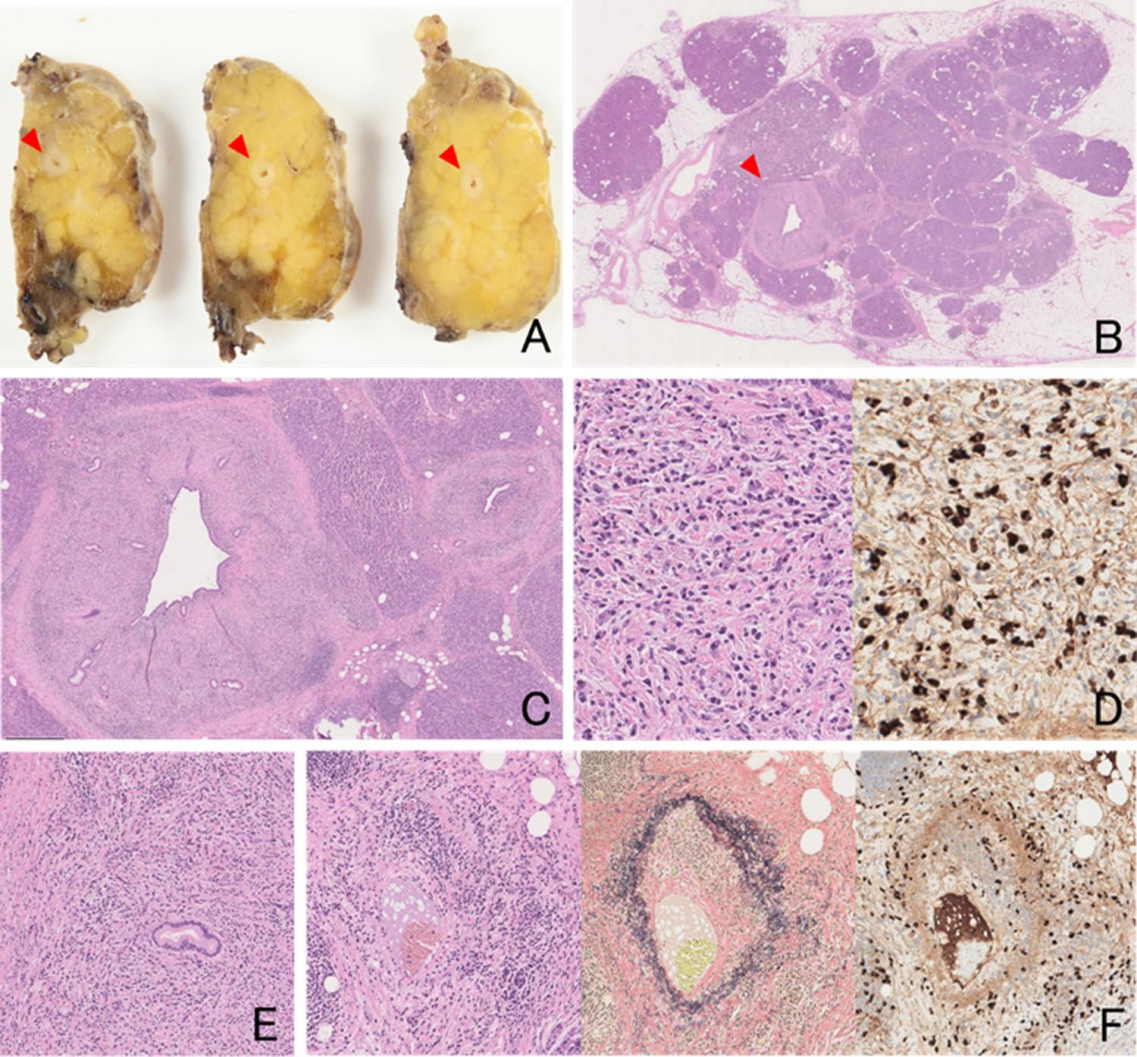
Gross and histopathological findings of the resected pancreatic specimen. **A** Gross appearance showing circumferential thickening of the main pancreatic duct (MPD) (red arrows). **B** Low-power view of the MPD (red arrows). **C** High-power view demonstrating marked fibrosis of the subepithelial layer in the MPD. **D** Dense lymphoplasmacytic infiltration and numerous IgG4-positive plasma cells (IgG4 immunostaining). **E** Storiform fibrosis observed in the inflammatory lesion. **F** Elastic fibers of the venous wall highlighted by EVG staining, with luminal narrowing caused by infiltration of IgG4-positive plasma cells (IgG4 immunostaining)

As cytology suggested potential malignancy, the patient underwent central pancreatectomy based on a preoperative diagnosis of suspected pancreatic CIS. Gross examination of the resected specimen revealed circumferential and uniform thickening of the MPD along its entire length. Histopathological findings showed marked fibrotic thickening of the subepithelial layer in the MPD, dense lymphoplasmacytic infiltration, > 50 IgG4-positive plasma cells per high-power field, and storiform fibrosis. In addition, obliterative phlebitis was observed, characterized by luminal narrowing of the veins due to fibrosis and dense infiltration of lymphocytes and plasma cells within the vessel walls (Fig. 5). Notably, the characteristic inflammatory and fibrotic changes were entirely confined to the subepithelial layer in the MPD, with no involvement of the surrounding pancreatic parenchyma. On detailed examination, only minimal fibrotic changes were detectable at the pancreatic margin and no malignant cells were observed. These findings were consistent with a diagnosis of AIP limited to the subepithelial layer in the MPD, based on the 2018 histopathologic criteria for AIP [1]. The patient was discharged on postoperative day 14. During postoperative follow-up, the serum IgG4 level was periodically monitored and remained persistently elevated, ranging from approximately 280–330 mg/dL. Despite this sustained elevation, the patient remained clinically stable for more than one year, with no evidence of pancreatic enlargement and no requirement for steroid therapy.

## Discussion

This case highlights a rare variant of AIP that posed diagnostic challenges. Unlike typical focal-type AIP, which generally manifests as a localized mass or pancreatic enlargement, our patient exhibited neither finding. The sole imaging abnormality was a short-segment stenosis of the MPD. Postoperative histological examination indicated that the inflammatory changes were strictly limited to the subepithelial layer in the MPD, with no involvement of the surrounding pancreatic parenchyma.

On imaging, AIP typically presents as diffuse or focal pancreatic enlargement. These findings reflect underlying histopathological changes, such as interstitial edema, fibrosis, and immune cell infiltration within the pancreatic parenchyma. Together, these alterations produce the characteristic swelling or mass-like lesions that can closely mimic PDAC [2]. Diffuse enlargement is typically linked to extensive pancreatic involvement, whereas focal-type AIP generally affects a specific area, manifesting as a localized mass or swelling. In contrast, our patient did not exhibit diffuse or focal pancreatic enlargement, although the resected specimen displayed definitive histological features of AIP. This atypical finding can be attributed to the characteristic inflammatory changes, such as fibrosis, lymphoplasmacytic infiltration, and obliterative phlebitis, being confined to the subepithelial layer in the MPD, without extending into the surrounding pancreatic parenchyma. Thus, this tissue expansion was insufficient to cause diffuse or focal swelling visible on imaging. Type 1 AIP typically exhibits peripancreatic inflammatory infiltration that forms a shell-like structure around the pancreatic parenchyma, corresponding to a capsule-like rim, which is observable on imaging [4]. In the present case, the minimal peripancreatic fibrotic changes were far less pronounced than this characteristic feature and insufficient to produce abnormalities detectable on imaging.

Previous reports have described focal-type AIP with segmental MPD stenosis; however, these cases consistently exhibited at least mild parenchymal swelling or mass formation [5, 6]. A thorough review of PubMed and other major databases identified no previously reported cases that reported the absence of both pancreatic swelling and mass formation and histological changes strictly limited to the periductal region of the MPD. To the best of our knowledge, the present study represents the first documented report of these characteristics. The pathophysiological basis for the duct-limited involvement observed in this case remains unclear. Given that the imaging findings were extremely atypical for AIP, several possibilities may be considered, including an extremely early stage of AIP confined to the MPD, residual ductal inflammation after spontaneous resolution of parenchymal involvement, or a previously unrecognized duct-predominant subtype. However, these hypotheses remain speculative, and clarification will require the accumulation of additional similar cases. If the present case had been managed conservatively, the duct-limited inflammatory changes might have subsequently progressed to involve pancreatic parenchyma and eventually produced the typical imaging features of AIP. Alternatively, spontaneous resolution of the localized inflammation might have occurred, resulting in improvement or disappearance of the isolated MPD stenosis. Nevertheless, these possibilities cannot be verified. Careful long-term follow-up is warranted, particularly given reports suggesting an increased risk of pancreatic cancer in patients with AIP.

Isolated MPD stenosis may raise concerns about pancreatic CIS, an early stage of pancreatic cancer that remains confined to the duct and does not form a detectable mass [7]. This makes the diagnostic process even more challenging, as a previous study reported that CIS may present solely as MPD stenosis with localized parenchymal atrophy, without any detectable mass formation [8]. In the present case, cytological analysis of pancreatic juice identified atypical cells, which suggested malignancy. Although the serum IgG4 level was mildly elevated, the increase was limited and insufficient to support a diagnosis of AIP, and the imaging findings were also atypical for AIP. Even if AIP had been considered in the differential diagnosis, a diagnostic steroid trial would not have been selected because pancreatic CIS could not be excluded. Therefore, surgical resection was performed because of the persistent suspicion of CIS.

Upon retrospective review of the preoperative imaging findings, several features were identified that were inconsistent with pancreatic CIS and instead compatible with AIP. First, localized pancreatic parenchymal atrophy was absent. Because focal atrophy is generally considered to precede or accompany upstream ductal dilatation and stenosis in CIS [9], the lack of this finding was regarded as inconsistent with CIS. Second, subtle ductal narrowing was also present in the pancreatic tail. However, this finding was not interpreted as clinically significant at the time of the preoperative evaluation, and only the focal stenosis in the pancreatic body was considered meaningful. Consequently, a preoperative diagnosis of suspected CIS was made, and central pancreatectomy was selected. If the ductal change in the pancreatic tail had been interpreted as significant, the relatively mild upstream ductal dilatation, together with the elevated serum IgG4 level, might have raised suspicion for AIP prior to surgery. Finally, the thickening of the subepithelial layer in the MPD observed on EUS was retrospectively considered to be compatible with AIP. This appearance closely resembles previously reported EUS and intraductal ultrasonography findings in IgG4-related sclerosing cholangitis, which are characterized by uniform submucosal wall thickening without epithelial abnormalities [10]. In addition, the band-like hypoechoic area surrounding the MPD corresponded histopathologically to IgG4-related inflammatory changes, while preservation of the innermost hyperechoic layer suggested an intact ductal epithelium. Collectively, these findings may have provided additional clues that could potentially have supported a preoperative suspicion of AIP, although establishing a definitive diagnosis based solely on these features would have remained challenging.

In considering the possibility of establishing a preoperative pathological diagnosis, the inflammatory and fibrotic changes were strictly confined to the subepithelial layer in the MPD, and therefore a definitive diagnosis using EUS-guided tissue acquisition would likely have been difficult. ERCP-guided intraductal biopsy of the pancreatic duct might also be considered as a possible diagnostic option, although its practical utility in this setting remains uncertain.

The ultimate diagnosis of AIP limited to the MPD accounts for the unusual imaging characteristics. This finding expands the known clinical and pathological spectrum of AIP and highlights the possibility of non-mass-forming, non-swelling presentations that closely mimic pancreatic CIS. Greater awareness of these atypical AIP presentations could enhance diagnostic accuracy and decrease unnecessary surgeries. The accumulation of cases will enhance our comprehensive understanding of this disease entity.
